# Buffalo Medical College

**Published:** 1849-05

**Authors:** 


*Buffalo Medical College.*—The annual return of the commencement, terminating the third session of the Buffalo Medical College, has suggested that some of the not inconsiderable number of readers of this Journal, added during the last two years, may desire to know more particulars of the history and prospects of the Institution, than have been gathered from our pages during the period just mentioned. We purpose, therefore, to give a brief sketch of its rise and progress, together with some account of the new College edifice, now in progress of completion. This Institution, although youthful in years, has attained a position (we may be permitted to say) which is in a high degree gratifying and encouraging to those who have been thus far identified with its history. It has had various obstacles to surmount, some of which have been of no ordinary character. We do not, however, design at this time, to consider these. We have no desire to perpetuate the recollection of them in the minds of those to whom they have been familiar, still less, to obtrude them upon the attention of others. Let them be forgotten, as we trust, they may be, ere long, by all!

The formal movement for the establishment of a medical College in this city, originated in the summer of 1846. It was at first intended to apply to the Legislature for a charter for a medical College exclusively, but it was afterwards concluded, at a convention of some of the most intelligent and influential citizens of the place, to make application for an University charter, embracing full powers to establish a medical department. The plan of a medical school in this city was not a novel one. The many advantages which Buffalo affords for such an Institution, had often suggested it, and projects had previously been entertained, which, however, it was never attempted to carry into execution.

The *University of Buffalo* was chartered during the Legislative session of 1846. Hon. Nathan K. Hall was the representative at the Assembly, at that time, and it was chiefly through his judicious, persevering efforts, that the charter was promptly obtained.

The medical department of the University (the only department as yet established) was organized in August, 1846. The present Faculty were then appointed, and the first lecture session was in the spring of 1847. Notwithstanding the short period between the issuing of the circular in the autumn of 1847, and the commencement of the term, the first class numbered more than sixty. The class assembled at the second session, in the spring of 1848, exceeded ninety in number. At the close of that session, it was concluded to adopt the plan proposed by the *National Medical Association, of a longer* term of instruction, and the session of 1848–9, which is just concluded, was accordingly extended to five months, to which was added a preliminary term of one month, chiefly devoted to dissections. Other recommendations by the National Association, not previously instituted, were adopted, the aim of the Faculty and Council, being to secure for the school a position which should entitle it to the respect and support of the profession. We believe we may say with all sincerity and truth, that the intrinsic and permanent character of the Institution, in every measure pursued to subserve its interests, has been held paramount to objects of a personal or selfish nature. The Buffalo College was the fourth, in the order of time, to extend the lecture term, and the only Institution northwest of the city of New York, that has adopted this change. One, only, of the two medical schools in the city of New York, has adopted it. The medical school of Boston, Mass., still adheres to the usual period, four months; while the several Institutions with which the Buffalo school, from proximity, must be brought, necessarily, more or less into competition, have not signified any intention of conforming to the recommendation of the Association. Under these circumstances, it has been resolved by the Buffalo Faculty, to return to the four months term, retaining, however, the preliminary term of one month, and adding daily lectures, on special subjects, to dissections during this term, making attendance not obligatory, but voluntary and gratuitous.

The lectures, dissections, &c., at this Institution, have heretofore been held at the building on the corner of Washington and Seneca streets, which was leased for a period of three years, for that purpose. Although it was extremely fortunate that a building was to be had, suitable for immediate necessities, it appeared important, in order to place the Institution upon a firm and permanent basis, that an edifice should be erected, adequate to prospective wants, and constructed with entire reference to purposes of medical instruction. For this end a subscription was commenced in the summer of 1847, and, in a short time, the sum of twelve thousand dollars was obtained, mostly in subscriptions of one hundred dollars, payable in four equal, semi-annual instalments. This, added to two thousand dollars appropriated by the State, was regarded as sufficient to authorize the commencement of a new edifice, which has been enclosed, is now in progress of completion, and will be in readiness for the session of 1849–50.

The ready and cheerful liberality which has been manifested by our citizens in the subscriptions for the erection of a College edifice will never be effaced from the grateful recollections of those connected with, and the immediate friends of, the school; nor will their names be forgotten as the first benefactors of the Institution, so long as it shall continue to exist. May the future history of the College afford more and more occasion for satisfaction to them, in the reflection that its success was, in a great measure, due to their timely and generous assistance!

The public spirit exhibited by these subscriptions is enhanced by the fact that they were made without any expectations of *pecuniary return*. The subscribers are stockholders to the amount of their subscriptions, but receive no dividends, nor interest. The stockholders, however, are the managers of the University, being the electors by whom the members of the Council are chosen. The present stockholders are mostly from the young business men of this city, than whom a more generous, enterprising, intelligent class, could with difficulty be found in any community. Their names are too many to be here enumerated. We may, however, without invidiousness, state that the first who enrolled his name as a subscriber was A. D. Patchin, Esq., attaching to his signature the sum of five hundred dollars. The largest subscription was by Jesse Ketchum, Esq., viz, six hundred dollars. Hon. Albert H. Tracy subscribed two hundred dollars; George W. Tifft, Esq., two hundred dollars; Hon. E. G. Spaulding, and Jabez Goodell, the same amount. About eighty subscribed one hundred dollars, and the balance was composed of subscriptions for sixty, and a few of forty dollars. These contributions were freely and promptly made, on a representation of the importance of the object for which they were designed, and we have no doubt, that should a further amount be required to be raised in the same way, an appeal would not be in vain. To the reader who resides in a larger and older city, it may, perhaps, seem that the liberality of our citizens, in this enterprise, is not so remarkable as to merit special eulogium. Instances of as great, or greater public generosity, are not, it is true, very infrequent. But, it is to be considered, in the first place, that the public have seldom exhibited much interest in, and disposition to contribute to the support of institutions for medical instruction. We doubt if another instance can be cited, in which the amount contributed by the citizens of Buffalo, has been raised in a similar manner. We do not say that similar instances may not have been, in this country, but we are not aware of there having been any. But, in the second place, many circumstances pertaining to a new city, like Buffalo, are to be taken into account. Our population, for the most part, is composed of young men, who come here to make up for want of pecuniary means, by enterprise and industry. We have not the men of wealth, and overplus of capital, which belong to older towns. The opportunities for business preponderate over the amount of money which might be profitably employed. Moreover, the necessary public expenditures in a new city, for laying out and paving streets, constructing sewers, and other improvements, render taxation heavy. Hence, the liberality of a contribution for an educational object, in which the contributors are not directly interested, is not to be measured by what may transpire in other places, where the circumstances just referred to, are widely different.

One of the advantages which this city presents for medical education consists in the opportunities which it affords for clinical illustrations of disease and surgery. For the two first sessions, these branches of practical instruction were chiefly confined to patients, presenting at a public dispensary for medical and surgical cases at the College. During the summer of 1848, however, a hospital was established, and placed under the direction of the Sisters of Charity, which soon was in successful operation. Fortunately for the interests of both Institutions, the building selected for the hospital, is situated but a few rods from the new College edifice. A proposition, on the part of the Faculty of the College, to render, gratuitously, medical and surgical services, was gladly accepted by the managers of the hospital, who, also, consented that, at stated times, under proper regulations, medical students might be admitted to visit the wards, in company with the attending physician and surgeon, on payment of a small fee, for the benefit of the hospital. This arrangement was at once entered into, and during the session just ended, highly valuable opportunities for witnessing surgical operations, the phenomena of disease, and the application of the principles of diagnosis and therapeutics, were afforded to those who chose to avail themselves of the hospital ticket.

The hospital has recently been placed on a broader and more permanent basis, by an appropriation of nine thousand dollars by the state Legislature, and the building, already adequate to receive one hundred patients, or more, is to be enlarged by the erection of another building, affording accommodations for nearly twice that number. All that could be desired, with respect to clinical instruction, is thus offered by this hospital. Indeed, as remarked by Prof's. Elliotson, Latham, and others, a hospital of moderate size, is better for the student, than one which is very large. A number of cases beyond what can be daily and carefully examined by the attending medical officers, and their history and progress, individually, followed by the student, proves a serious obstacle in the way of both teacher and pupil. The attention is overtasked and confused, to the inconvenience of all parties, inclusive of the patients. It is not the aggregate number of cases that are seen, that renders hospital observation valuable, but the care and attention which are bestowed upon their study. A hospital with an hundred, or hundred and fifty beds, if it receive a sufficient variety of cases, is sufficient for all the purposes of clinical instruction, and, in our opinion, much increase beyond this size, is to be regretted, rather than desired.

The remainder of this article will be devoted to a description of the College edifice, condensed from an article which appeared several weeks ago, in one of the daily papers of this city.

The building is situated on the corner of Virginia and Main streets, at a pleasant part of the city, somewhat elevated, and sufficiently removed from the centre of business, to be free from the noise and disturbance of the latter. It is one hundred feet long, by fifty-six wide, and four stories high. The style of architecture is Norman, or, more properly, Romanesque, a style regarded as peculiarly appropriate for buildings designed for similar purposes. The windows are arched. The walls are of the Lockport red sand, or free stone, resembling the New Jersey red stone, of which Trinity church, N. Y., is built. They present a rough, unhewn surface, the effect of which is to give an appearance of massiveness, age, and strength to the building, and accords with the style of architecture adopted.

The Faculty of the College, before determining on any plan to recommend to the Council, sent a delegate to New York and Philadelphia, to make personal examinations of the College buildings, and to obtain accurate admeasurements. From the information thus collected, plans were digested, which, it is believed, will render the building one of the most convenient structures for the purposes for which it is designed, in the country. The plans were executed by the talented artist, C. N. Otis Esq., now in Europe.

The centre of the building is devoted to two large rooms, the anatomical amphitheatre, and College Hall, or general lecture room. The width of each is forty feet, and the length fifty feet. The height of one is twenty, and of the other thirty feet. Each will seat, comfortably, from three hundred and fifty, to four hundred persons.

In front, the first floor on each side of the hall, is a room for office and library, a little more than twenty feet square.

The three remaining stories in front, to the extent of the entire width of the building, (fifty feet) are appropriated to a general and anatomical museum. It is to be galleried, and to run up to the roof, which is to be paneled. In the centre of the roof is to be a sky-light, with stained glass. This plan provides most ample room for an extensive museum, and will present a very beautiful effect.

In the rear of the building, on the first floor, and connected with the general lecture room, are large rooms for the convenience of the lecturer on chemistry, laboratory, and a private room for other Professors.

On the second floor, in the rear, are several rooms for the use of the Professors.

The rear of the two upper stories, embracing the entire width of the building, is devoted to dissecting rooms.

The basement embraces apartments for the accommodation of the Janitor.

The work of completing the building is now in rapid progress, and the lectures for the session of 1849–50, Providence permitting, will be therein delivered.

